# The Association between PON1 (Q192R and L55M) Gene Polymorphisms and Risk of Cancer: A Meta-Analysis Based on 43 Studies

**DOI:** 10.1155/2019/5897505

**Published:** 2019-07-28

**Authors:** Xiaolan Pan, Lei Huang, Meiqin Li, Dan Mo, Yihua Liang, Zhiming Liu, Zhaodong Huang, Lingsha Huang, Jinfeng Liu, Bo Zhu

**Affiliations:** ^1^Department of Clinical Laboratory, The Affiliated Tumor Hospital of Guangxi Medical University, Nanning, 530021 Guangxi, China; ^2^Department of Surgery, Maternal and Child Health Hospital of the Guangxi Zhuang Autonomous Region, Nanning, China

## Abstract

Q192R and L55M polymorphism were considered to be associated with the development of multiple cancers. Nevertheless, the results of these researches were inconclusive and controversial. Therefore, we conducted a meta-analysis of all eligible case-control studies to assess the association between PON1 (Q192R and L55M) gene polymorphisms and risk of cancer. With the STATA 14.0 software, we evaluated the strength of the association by using the odds ratios (ORs) and 95% confidence intervals (CIs). A total of 43 case-control publications 19887 cases and 23842 controls were employed in our study. In all genetic models, a significant association between PON1-L55M polymorphisms and overall cancer risk was observed. Moreover, in the stratified analyses by cancer type, polymorphism of PON1-L55M played a risk factor in the occurrence of breast cancer, hematologic cancer, and prostate cancer. Similarly, an increased risk was observed in the Caucasian and Asian population as well as hospital-based group and population-based group. For PON1-Q192R polymorphisms, in the stratified analyses by cancer type, PON1-Q192R allele was associated with reduced cancer risks in breast cancer. Furthermore, for racial stratification, there was a reduced risk of cancer in recession model in Caucasian population. Similarly, in the stratification analysis of control source, the overall risk of cancer was reduced in the heterozygote comparison and dominant model in the population-based group. In conclusion, PON1-Q192R allele decreased the cancer risk especially breast cancer; there was an association between PON1-L55M allele and increased overall cancer risk. However, we need a larger sample size, well-designed in future and at protein levels to confirm these findings.

## 1. Introduction

Cancer is one of the diseases caused by a combination of genetic and environmental factors [1]. The PON1 gene, located on the long arm of chromosome 7q21.3, is an antioxidant enzyme that has strong lipophilic antioxidant properties, which can maintain the balance of antioxidant-oxidant [2, 3]. Simultaneously, PON1 is also an esterase involved in scavenging reactive oxygen species by binding to high-density lipoprotein (HDL). Studies have shown that oxidative stress may participate in the process of cell proliferation and malignant transformation and damage DNA as well as other biological molecules, resulting in the occurrence of tumors [4]. The ability of PON1 detoxification of carcinogenic oxidative stress products makes it possible for researchers to predict PON1 gene polymorphism in cancer susceptibility [5].

At present, with the deep development of genetic studies of PON1, studies have found that PON1-Q192R and PON1-L55M, the two most common functional genetic polymorphisms in PON1, were identified at positions 192 and 55 [6]. PON1-Q192R polymorphism (rs662A > G) was caused by the glutamine (Q genotype) substituted for the arginine (R genotype) 192 of the gene 6 exon of the PON 1 gene [7]. PON1-L55M (rs854560) was originated from the replacement of 55 leucines (L genotype) by methionine (M genotype) at third exon 55[8]. In addition, it has been shown that the two functional SNP, Q192R and L55M, were associated with the risk of multiple tumors [9, 10], such as oral cancer [11], lung cancer [12], and embryonal tumors [13].

According to the important role of PON1 in the development of tumor and the correlation between genotype and phenotype, we speculate that the variation of PON1 gene Q192 R and L55M may be related to tumor susceptibility. However, the data of many studies are contradictory and uncertain at present. Therefore, a comprehensive meta-analysis should be conducted to determine the relationship between Q192R and L55M polymorphism and cancer risk.

## 2. Materials and Methods

### 2.1. Search Strategy

We conducted a systematic literature search in the PubMed, Embase, and Web of Science for all related studies before June 10, 2019 via utilizing the following terms: “polymorphism OR paraoxonase 1 OR PON1” AND “tumor OR malignancy OR cancer OR carcinoma OR neoplasm”. In addition, we extracted the reference of the original articles on this issue to carry out a hand search for extra studies. The results deduced from these articles were limited to humans. When the publication referred to more than one cancer type or ethnicity, we deled with data respectively. Besides, if different authors published articles based on the same population or one author used similar data in an article, we picked out the report with the latest study and largest sample size.

#### 2.1.1. Inclusion Criteria and Exclusion Criteria

The enrolled studies must contain the following inclusion criteria: (1) publication that evaluated the association between PON1-L55M, or PON1-Q192R polymorphism and the risk of cancer. (2) The genotype frequency may be obtainable from cases and controls, or we could gain it via computing. In addition, studies were excluded when they would meet these exclusion criteria: (1) reviews, case reports, or case-only studies; (2) studies with deficient genotype frequency date; (3) animals reports; and (4) replicate studies.

### 2.2. Data Extraction

The authors were able to excerpt relevant data from these qualified studies independently, and the following information would be seized: first author's last name, publishing year, the ethnicity of each population, the genotyping methods, the control of source, cancer types, number of cases and controls, and P value of Hardy–Weinberg equilibrium. When encountering divergences, we analyzed the report and reached a consistent agreement lastly.

### 2.3. Statistical Analysis

95% confidence interval (CI) and odds ratio (OR) were utilized to estimate the relation between PON1-Q192R, or PON1-L55M polymorphism and the risk of cancer with five genetic models: heterozygote comparison (ML versus LL; RQ versus QQ), allele contrast (M versus L; R versus Q), homozygote (MM versus LL; RR versus QQ), recessive (MM versus ML+LL; RR versus RQ+QQ), and dominant (ML+MM versus LL; RR+RQ versus QQ). Besides, stratified analyses were conducted via ethnicity, cancer type, control source, and genotyping method. However, when any cancer type is less than two studies, we would segment it into the “other cancers” group. In addition, *χ*^2^-test-based* Q-*statistic test [14] was taken to assess the research heterogeneity while *I*^2^ values and* P *values [15] were used for quantifying. When I^2^< 50% and* P*>0.10, it indicates that there was no significant heterogeneity, and ORs could be pooled by a fixed-effects model. Otherwise, the random effects model would be adopted [16]. Furthermore, sensitivity analysis, from the qualified removing a single research study and revealing the individual data set to merge OR influence, was applied to estimate the stability of these data. (*P*<0.05 was regarded as statistically significant [17].) Finally, potential publication bias was estimated by symmetry of funnel plot of Begg's test as well as Egger's test [15, 18], and being statistically significant was considered when P<0.05. All statistical tests were performed with STATA Software (version 14.0, state Corp), and* P*<0.05 for any genetic models or tests was identified as statistically significant.

## 3. Result

### 3.1. Publication Characteristics

**Figure 1:**
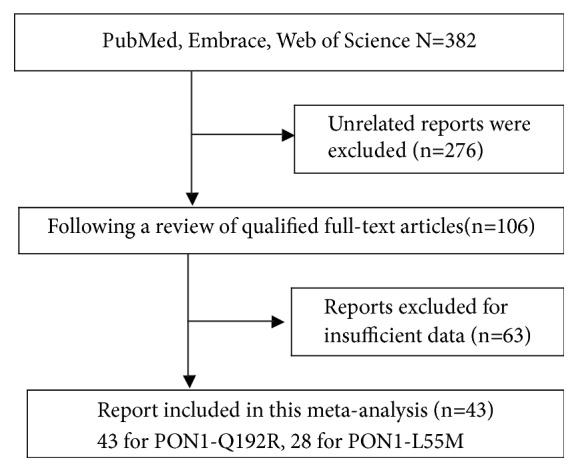
Flow chart of the report selection process.

According to the inclusion criteria after detailed examination, a total of 43 case-control publications including 19977 cases and 23932 controls were employed in our study [11–13, 19–59]. The flow chart of the study screening process was summarized in Figure 1. Moreover, there were 43 studies with 11412 cases and 13936 controls for PON1-Q192R polymorphism (Table 1), and, for* PON1 *L55M polymorphism, 28 studies involved a total of 8565 cases and 9996 controls (Table 2). For* PON1 *Q192R polymorphism, a total of 8 cancer types were processed, including breast cancer [21, 27, 31, 32, 37, 39, 50], prostate cancer [22, 23, 40, 41], gastrointestinal cancer [19, 20, 48, 59, 60], hematologic tumor [25, 29, 33, 44], lung cancer [11, 12, 54], brain tumors [30, 35, 38, 45, 56, 57], ovarian cancer [34, 43] and other cancers [13, 26, 28, 42, 53, 58] (uterine leiomyoma, childhood embryonal tumors, metastatic gastric cancer, bladder cancer, and renal cell cancer). Besides, we disposed a total of 7 cancer types when dealing with PON1-L55M polymorphism nearly like PON1 Q192R polymorphism. In addition, For* PON1 *Q192R polymorphism, 9 publications were conducted in Asians, 9 in mixed group, and 25 publications in Caucasians. Besides, there were 15 studies divided by TaqMan assay, while 28 studies conducted by PCR- RFLP. Moreover, the majority of control groups in the case group are gender and age matching, including 23 hospital based and 20 population based. For* PON1 *L55M polymorphism, we also conducted 6, 6, and 16 studies in Asian, mixed group, Caucasians, respectively. Moreover, 10 studies were divided by TaqMan assay as well as 18 studies conducted by PCR- RFLP.

### 3.2. Meta-Analysis

#### 3.2.1. Association between PON1-Q192R and Cancer Susceptibility

**Figure 2:**
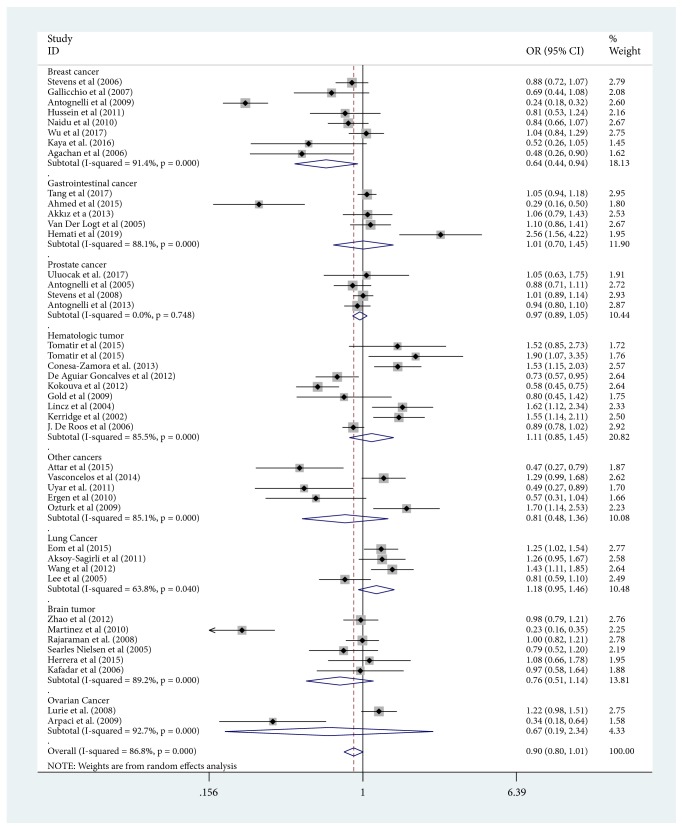
Meta-analysis of the association between PON1-Q192R polymorphism and cancer risk (R versus Q). Abbreviations: ID, identification; CI, confidence interval; NA, not available; OR, odds ratio; weights come from random effects analysis.

In summary, in allele contrast model, we have found that there were not association between PON1-Q192R allele and reduced overall cancer risk (Table 3). In the subgroup analysis of cancer type, we identified a decreased risk in breast cancer (R versus Q: OR=0.643, 95%CI=0.440-0.942; RR versus QQ: OR=0.542, 95%CI=0.331-0.886; RQ versus QQ: OR=0.529, 95%CI=0.325-0.861; and RR+RQ versus QQ: OR=0.534, 95%CI=0.330-0.865). Nevertheless, an increased risk was confirmed in prostate cancer in the dominant model (RR+RQ versus QQ: OR=1.249, 95%CI=1.030-1.514). Furthermore, by racial stratification, there was a reduced risk of cancer in recession model (RR+RQ versus QQ: OR=0.744, 95%CI=0.557-0.993) among Caucasian population. Similarly, in the stratification analysis of control source, the overall risk of cancer is reduced in the heterozygote comparison and dominant model (RQ versus QQ: OR= 0.793, 95%CI=0.638-0.984; RR+RQ versus QQ: OR=0.789, 95%CI=0.630-0.988) in the population-based group. In addition, we did not observe any risk factor by stratified analysis using genotyping method.  Figure 2 showed the meta-analysis of the association between PON1-Q192R polymorphism and cancer risk (R versus Q)

#### 3.2.2. Association between PON1-L55M and Cancer Susceptibility

**Figure 3:**
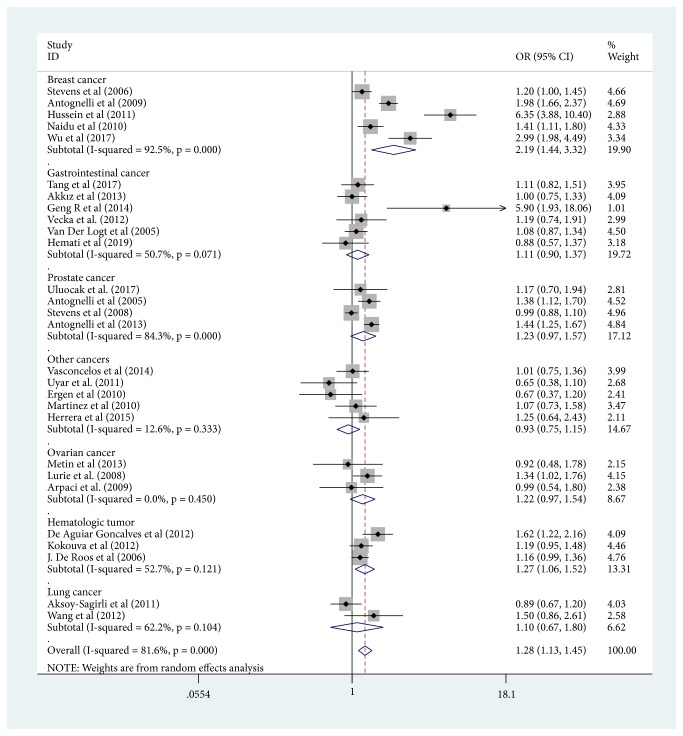
Meta-analysis of the association between PON1-L55M polymorphism and cancer risk (M versus L). Abbreviations: ID, identification; CI, confidence interval; NA, not available; OR, odds ratio; weights come from random effects analysis.

Our study had uncovered that the PON1-L55M polymorphism was significantly associated with an increased risk of the overall cancers under all the genetic models (Table 4) (M versus L: OR =1.277, 95% CI =1.127-1.448; MM versus LL: OR =1.507, 95% CI =1.205-1.885; ML versus LL: OR =1.192, 95%CI =1.064-1.337; MM versus ML+LL: OR =1.288, 95%CI =1.120-1.408; ML+MM versus LL: OR =1.417, 95%CI =1.176-1.708). Furthermore, we found an increased risk of breast cancer under all the five models when conducting the cancer type subgroup analysis (M versus L: OR =2.186, 95%CI =1.438-3.323; MM versus LL: OR =3.215, 95%CI=1.756-5.886; ML versus LL: OR =1.579, 95%CI=1.145-2.177; MM versus ML+LL: OR =2.727, 95%CI=1.563-4.756; ML+MM versus LL: OR =2.110, 95%CI =1.397-3.188), prostate cancer in the dominant and heterozygote comparison model (ML versus LL: OR =1.291, 95% CI =1.071-1.557; ML+MM versus LL: OR =1.341, 95%CI=1.024-1.756), and hematologic tumor in the allele contrast model (M versus L: OR =1.271, 95% CI =1.059-1.525), homozygote model (MM versus LL: OR =1.514, 95%CI =1.178-1.946), recessive model (MM versus ML+LL: OR =1.405, 95%CI =1.111-1.778), and dominant model (ML+MM versus LL: OR =1.299, 95%CI =1.017-1.661).  Figure 3 showed the meta-analysis of the association between PON1-L55M polymorphism and cancer risk (M versus L).

Similarly, an increased risk was observed in the Caucasian population under the five genetic models: M versus L: OR =1.231, 95% CI = 1.028-1.474; MM versus LL: OR =1.737, 95% CI =1.519-1.986; ML versus LL: OR =1.170, 95% CI =1.034-1.324; MM versus ML+LL: OR =1.407, 95%CI =1.092-1.813; ML+MM versus LL: OR =1.334, 95%CI =1.215-1.465, the Asian population (M versus L: OR =1.604, 95% CI =1.089-2.363; MM versus LL: OR =2.093, 95% CI =1.295-3.381; ML versus LL: OR =1.550, 95% CI =0.995-2.417; MM versus ML+LL: OR =1.624, 95%CI =1.041-2.535; ML+MM versus LL: OR =1.967, 95%CI =1.238-3.125), the mixed population (M versus L: OR =1.177, 95%CI =1.004-1.379; ML+MM versus LL: OR =1.126, 95%CI =1.006-1.261) (Table 4), hospital-based group (M versus L: OR =11.240, 95%CI=1.056-1.456; MM versus LL: OR =1.531, 95%CI =1.199-1.955; ML versus LL: OR =1.255, 95%CI =1.020-1.543; MM versus ML+LL: OR =1.288, 95%CI =1.120-1.480; ML+MM versus LL: OR =1.411, 95%CI=1.173-1.698), and population-based group (M versus L: OR =1.325, 95%CI=1.085-1.618; MM versus LL: OR =1.568, 95%CI =1.091-2.253; ML versus LL: OR =1.275, 95%CI =1.051-1.548; MM versus ML+LL: OR =1.503, 95%CI =1.110-2.034; ML+MM versus LL: OR =1.222, 95%CI=1.122-1.331). In addition, we identified an increased risk by stratified analysis using genotyping method.

#### 3.2.3. Publication Bias and Sensitivity Analysis

**Figure 4:**
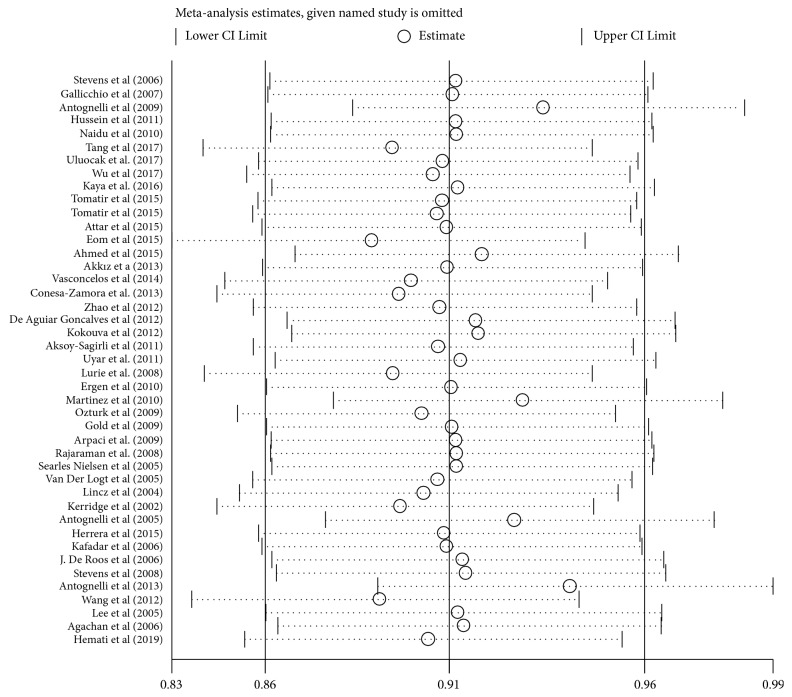
Sensitivity analysis of PON1-Q192R in overall OR coefficients (RR versus QQ). Abbreviations: OR, odds ratio CI, confidence interval. Sequentially calculated results of each study are omitted. Both ends of the broken line represent 95% of the CI.

**Figure 5:**
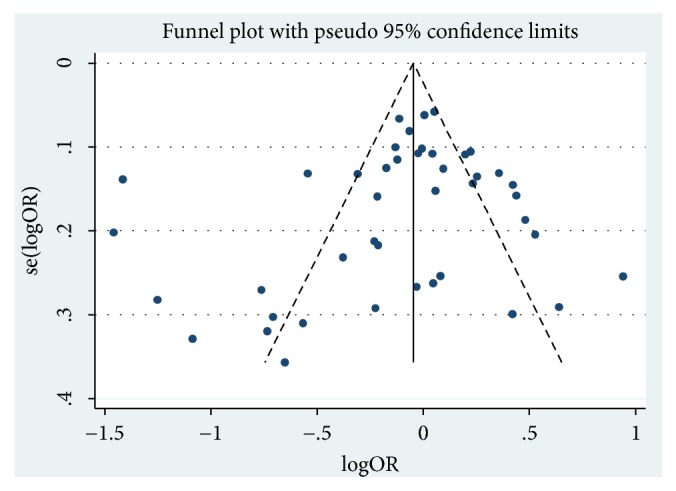
Funnel figure of PON1-Q192R in overall OR coefficients (RR versus QQ). Abbreviations: OR, odds ratio.

A sensitivity analysis was carried out to detect the impact of individual papers on whole data by getting rid of one report at a time from the pooled analysis. And no individual report has been significantly affected by the pooled OR.  Figure 4 showed the plot of the sensitivity analysis for evaluating the association between PON1-Q192R and cancer risk (RR versus QQ). Besides, we perform Egger's test and Begg's funnel plot to evaluate publication bias (Figure 5). And the results of Egger's test and Begg's funnel plot did not uncover publication bias in PON1 (Q192R and L55M) gene polymorphisms (PON1 Q192R: R versus Q: Begg's test: z=1.74 P=0.082; Egger's test: t= -1.26 P=0.216; PON1-L55M: M versus L: Begg's test: z=0.06 p=0.953; Egger's test: t= 0.66; P=0.516). Thus, our results are believable due to the absence of significant publication bias in our meta-analysis.

## 4. Discussion

Several studies have indicated that PON1, which is one of xenobiotic metabolising enzymes, plays a crucial role in the detoxification of carcinogenic compounds and decreases oxidative stress. Genetic polymorphisms can influence the enzyme and modify its activity, resulting in an impact on individual sensitivity to certain pathologies [61]. Indeed, a great deal of researches have showed that polymorphisms encoding the gene of these enzymes have been linked to the progression of cancer [49, 62]. Furthermore, several variants of PON1, including Q192R and L55M, have been found to be a biologically reasonable candidate which has an obvious influence on cancer. PON1 (Q192R and L55M) gene polymorphisms were related to many types of cancer, such as breast, prostate, and hepatocellular carcinoma [20, 50, 63]. For instance, PON1-L55M polymorphism may increase the risk in multiple cancer types, such as prostate and breast cancers but decrease renal cell carcinoma and ovarian cancer risk. As for PON1-Q192R, it has been revealed to suppress expression in lung [64] and pancreatic cancer [65] and reduce the risk of breast and prostate cancers. And the results of these researches were inconclusive and controversial.

In our work, in all genetic models we have identified the significant association between PON1-L55M polymorphism and overall cancer risk, while PON1-Q192R allele was not associated with reduced overall cancer risks. In the stratified analysis, we observed an increased risk in the Caucasian population and the Asian population, as well as the hospital-based group and population-based group under all the five genetic models in the PON1-L55M polymorphism. Similarly, a significantly increased risk of the overall cancers under the homozygote, allele contrast, recessive, and dominant models was uncovered in hematological tumor in the PON1-L55M polymorphism. Nevertheless, in the PON1-Q192R polymorphism, we also observe a reduced risk of the overall cancers in the allele contrast and dominant models. Meanwhile, we could obtain an interesting phenomenon that PON1-L55M polymorphism acts as a risk factor in all the five genetic models and there was an association between Q192R polymorphism and a reduced risk for cancer progression (except recessive model) after stratified analyses by cancer type, especially breast cancer. Thus, we can obtain that PON1 (Q192R and L55M) gene polymorphisms play a vital role in the development of breast cancer, whose mechanism maybe as follows: there was a critical association between L allele and higher PON1 serum concentrations while M variant decreased the stability of this enzyme. Therefore, the blood concentration of PON1 was reduced in this way; then, the activity of the enzyme was influenced, which may increase the vulnerability to genomic damage by reducing the inflammatory oxidant and the detoxifying ability of dietary carcinogens, thereby increasing the risk of breast cancer [5]. Furthermore, breast cancer becomes more susceptible to genomic damage as a result of lower levels of PON1 which could decrease the ability to detoxify inflammatory oxidants and dietary carcinogens [5]. Similarly, the exchange of Q and R could produce an enzyme which has a higher detoxification activity when there were potential carcinogenic products of oxidative stress and lipid peroxidation [66, 67]. In addition, not only genetic factors but also other contributors including nutrition and lifestyle can significantly affect PON1 enzyme activity, thereby reducing the risk of breast cancer [68]. To sum up, PON1, as a member of lipid peroxidation scavenging systems, may have an impact on malignant transformation and cell proliferation in the progression of breast cancer [69]. In the ethnographic analysis, we found ethnic groups having different results, which may be due to ethnic living habits, living environment, and genetic factors.

Previous meta-analysis also reported the association of PON1 polymorphism with cancer risk [10, 70]. As far as we know, we are the first of the typical functional polymorphism of the PON1 gene including all the published and defined case-control studies that have been conducted in a comprehensive meta-analysis. Compared with previous researches, our report was more persuasive and we have carried out a more detailed analysis to demonstrate our results. First and most obviously, the data we collected in our study was up-to-date, and we could keep up with the research front. Secondly, we included more qualified studies and larger sample size, which indicates that we are relatively more accurate in assessing that association between the PON1 gene SNPs and the risk of cancer.

Despite the association between PON1 (Q192R and L55M) gene polymorphism and cancer risk which has been studied in detail, we should note some limitations at the same time. First of all, the quantity of publications collected in our study was limited and there was a relatively small sample size of the report. What is more, Caucasian accounted for the most of the registered publications and there were no Africans. Furthermore, some of publications would only publish positive results, which could make the meta-analysis less credible. Lastly, our results were based on the estimates of single-factor, which could lead to serious confusion and bias due to the lack of raw data, and there is a need to adjust the effect size with possible confounders related to lifestyle risk factors, such as age, obesity, alcohol consumption, and smoking.

In conclusion, our study has demonstrated that PON1-Q192R can significantly reduce the risk of cancer and the polymorphism of PON1-L55M is a risk factor leading to cancer, especially breast cancer. Next, we need a larger sample size at protein levels to confirm whether PON1 polymorphisms may be potential genetic markers of tumor prognosis and identify its role in the risk of women developing breast cancer.

## Figures and Tables

**Table 1:** Characteristics of qualified case-control studies included in the meta-analysis of PON1-Q192R.

Author	Year	Ethnicity	Genotyping Method	Control of source	Cancer Type	Case			Control			HWE		
						QQ	QR	RR	QQ	QR	RR	**χ** ^2^	p	p(HWE)
Stevens et al.	2006	Caucasian	PCR-RFLP	P-B	Breast Cancer	259	182	42	238	198	47	0.38	0.54	Y
Gallicchio et al.	2007	Caucasian	PCR-RFLP	P-B	Breast Cancer	38	15	5	469	353	82	1.93	0.19	Y
Antognelli et al.	2009	Caucasian	PCR-RFLP	P-B	Breast Cancer	484	50	13	340	152	52	27.19	0.00	N
Hussein et al.	2011	Caucasian	PCR-RFLP	P-B	Breast Cancer	51	41	8	46	42	12	0.25	0.62	Y
Naidu et al.	2010	Asian	PCR-RFLP	H-B	Breast Cancer	200	158	29	115	115	22	0.81	0.37	Y
Tang et al.	2017	Asian	TaqMan	P-B	Esophagogastric	408	501	132	691	776	207	0.23	0.63	Y
Uluocak et al.	2017	Caucasian	PCR-RFLP	H-B	Prostate Cancer	24	17	8	45	42	11	0.06	0.80	Y
Wu et al.	2017	Asian	TaqMan	H-B	Breast Cancer	155	156	54	167	156	55	3.42	0.06	Y
Kaya et al.	2016	Caucasian	TaqMan	H-B	Breast Cancer	10	11	11	5	13	17	0.88	0.35	Y
Tomatir et al.	2015	Caucasian	PCR-RFLP	H-B	Hematologic Cancer	36	20	4	58	24	2	0.07	0.79	Y
Tomatir et al.	2015	Caucasian	PCR-RFLP	H-B	Hematologic Cancer	33	21	6	58	24	2	0.07	0.08	Y
Attar et al.	2015	Caucasian	PCR-RFLP	H-B	Uterine Leiomyoma	60	8	8	50	47	6	1.39	0.24	Y
Eom et al.	2015	Asian	PCR-RFLP	H-B	Lung Cancer	37	170	209	48	188	180	0.01	0.92	Y
Ahmed et al.	2015	Asian	PCR-RFLP	P-B	Colorectal Cancer	30	16	4	20	36	24	0.76	0.38	Y
Akkız et al.	2013	Caucasian	PCR-RFLP	P-B	Hepatocellular Carcinoma	109	95	13	115	88	14	0.27	0.60	Y
Vasconcelos et al.	2014	Mixed	TaqMan	H-B	Embryonal Tumors	36	85	41	104	160	72	0.51	0.48	Y
Conesa-Zamora et al.	2013	Caucasian	TaqMan	H-B	Lymphomas	83	99	33	100	104	10	7.00	0.01	N
Zhao et al.	2012	Asian	TaqMan	H-B	Glioma	161	158	52	159	167	52	0.59	0.44	Y
De Aguiar Goncalves et al.	2012	Mixed	TaqMan	H-B	Hematologic Tumor	96	102	40	74	106	54	1.790	0.180	Y
Kokouva et al.	2012	Caucasian	PCR-RFLP	H-B	Hematologic Cancer	213	88	15	181	141	29	0.04	0.83	Y
Aksoy-Sagirli et al.	2011	Caucasian	PCR-RFLP	H-B	Lung Cancer	93	111	19	121	93	20	0.13	0.72	Y
Uyar et al.	2011	Caucasian	PCR-RFLP	P-B	Renal Cell Cancer	38	21	1	27	27	6	0.04	0.84	Y
Lurie et al.	2008	Mixed	TaqMAN	P-B	Ovarian Cancer	66	120	86	122	211	111	1.07	0.30	Y
Ergen et al.	2010	Caucasian	PCR-RFLP	H-B	Osteosarcoma	27	21	2	15	33	2	0.06	0.80	Y
Martinez et al.	2010	Caucasian	TaqMan	H-B	Brain Tumor	31	33	9	22	89	109	0.37	0.54	Y
Ozturk et al.	2009	Caucasian	PCR-RFLP	H-B	Bladder Cancer	8	53	15	37	84	14	10.71	<0.001	N
Gold et al.	2009	Mixed	PCR-RFLP	P-B	Multiple Myeloma	10	19	13	9	27	19	0.01	0.91	Y
Arpaci et al.	2009	Caucasian	PCR-RFLP	H-B	Ovarian Cancer	38	6	6	17	29	6	1.46	0.23	Y
Rajaraman et al.	2008	Mixed	TaqMan	H-B	Brain Tumor	266	207	39	244	165	44	4.10	0.04	N
Searles Nielsen et al.	2005	Mixed	TaqMan	P-B	Brain Tumor	32	28	6	100	105	31	0.17	0.68	Y
Van der Logt et al.	2005	Caucasian	PCR-RFLP	P-B	Colorectal Cancer	180	150	24	158	120	17	0.87	0.35	Y
Lincz et al.	2004	Caucasian	PCR-RFLP	P-B	Multiple Myeloma	33	41	16	103	74	22	2.35	0.13	Y
Kerridge et al.	2002	Caucasian	PCR-RFLP	P-B	Lymphoma	73	50	39	103	74	22	2.35	0.13	Y
Antognelli et al.	2005	Caucasian	PCR-RFLP	H-B	Prostate Cancer	197	168	20	212	85	64	67.85	<0.001	N
Herrera et al.	2015	Mixed	TaqMan	H-B	Brain Tumor	15	32	20	12	32	14	0.64	0.42	Y
Kafadar et al.	2006	Caucasian	PCR-RFLP	H-B	Brain Tumor	43	26	15	24	18	8	1.96	0.16	Y
J. De Roos et al.	2006	Mixed	TaqMan	P-B	Hematologic Cancer	540	453	127	415	403	117	1.53	0.22	Y
Stevens et al.	2008	Mixed	TaqMan	P-B	Prostate Cancer	624	537	95	656	487	121	4.74	0.03	N
Antognelli et al.	2013	Caucasian	PCR-RFLP	H-B	Prostate Cancer	291	250	30	707	258	203	244.08	<0.001	N
Wang et al.	2012	Asian	PCR-RFLP	P-B	Lung Cancer	36	177	143	38	84	62	0.93	0.33	Y
Lee et al.	2005	Asian	TaqMan	P-B	Lung Cancer	24	80	73	11	89	77	4.999	0.025	N
Agachan et al.	2006	Caucasian	PCR-RFLP	P-B	Breast Cancer	17	4	12	6	29	17	1.461	0.230	Y
Hemati et al.	2019	Asian	PCR-RFLP	H-B	Gastric Cancer	39	41	10	62	26	2	0.03	0.87	Y

*Abbreviations*: PCR-RFlP, polymerase chain reaction-restriction fragment length polymorphism; HWE, Hardy–Weinberg equilibrium; Y, polymorphisms conforming to HWE in the control group; N, polymorphisms not conforming to HWE in the control group; H-B, hospital based; P-B, population based.

**Table 2:** Characteristics of qualified case-control studies included in the meta-analysis of PON1- L55M.

Author	Year	Ethnicity	Genotyping Method	Control of source	Cancer Type	Case			Control			HWE		
						LL	LM	MM	LL	LM	MM	**χ** ^2^	p	p(HWE)
Stevens et al.	2006	Caucasian	PCR-RFLP	P-B	Breast Cancer	176	230	77	202	233	58	0.88	0.77	Y
Antognelli et al.	2009	Caucasian	PCR-RFLP	P-B	Breast Cancer	107	115	325	188	125	231	157.2	0.0001	N
Hussein et al.	2011	Caucasian	PCR-RFLP	P-B	Breast Cancer	19	21	60	35	23	6	0.58	0.44	Y
Naidu et al.	2010	Asian	PCR-RFLP	P-B	Breast Cancer	159	178	50	126	109	17	1.04	0.308	Y
Tang et al.	2017	Asian	TaqMan	P-B	Esophagogastric Cancer	971	69	1	1573	99	2	0.12	0.73	Y
Uluocak et al.	2017	Caucasian	PCR-RFLP	H-B	Prostate Cancer	19	24	6	43	45	10	0.13	0.72	Y
Wu et al.	2017	Asian	TaqMan	H-B	Breast Cancer	284	72	9	346	30	2	3.24	0.064	Y
Akkız et al.	2013	Caucasian	PCR-RFLP	P-B	Hepatocellular Carcinoma	105	81	31	101	89	27	1.12	0.29	Y
Geng R et al.	2014	Asian	TaqMan	H-B	Metastatic Gastric Cancer	11	7	0	82	7	0	0.15	0.7	Y
Vasconcelos et al.	2014	Mixed	TaqMan	H-B	Embryonal Tumors	85	56	15	177	134	25	0.023	0.95	Y
Metin et al.	2013	Caucasian	PCR-RFLP	H-B	Ovarian Cancer	33	22	0	33	19	2	0.13	0.72	Y
Vecka et al.	2012	Caucasian	PCR-RFLP	H-B	Pancreatic Cancer	24	39	10	28	37	8	0.67	0.41	Y
De Aguiar Goncalves et al.	2012	Mixed	TaqMan	H-B	Acute Leukemia	104	99	34	131	75	19	2.91	0.09	Y
Kokouva et al.	2012	Caucasian	PCR-RFLP	H-B	Hematologic Cancer	117	139	60	142	159	50	0.26	0.61	Y
Aksoy-Sagirli et al.	2011	Caucasian	PCR-RFLP	H-B	Lung Cancer	119	94	10	118	102	14	1.75	0.19	Y
Uyar et al.	2011	Caucasian	PCR-RFLP	P-B	Renal Cell Cancer	29	25	6	21	29	10	4.96	0.998	Y
Lurie et al.	2008	Mixed	TaqMan	P-B	Ovarian Cancer	14	65	192	24	145	276	0.74	0.39	Y
Ergen et al.	2010	Caucasian	PCR-RFLP	H-B	Osteosarcoma	24	23	3	21	20	9	1.14	0.29	Y
Martínez et al.	2010	Caucasian	TaqMan	H-B	Brain Tumor	11	32	30	38	94	88	2.15	0.14	Y
Arpaci et al.	2009	Caucasian	PCR-RFLP	H-B	Ovarian Cancer	27	19	5	25	27	2	2.65	0.103	Y
Van der Logt et al.	2005	Caucasian	PCR-RFLP	P-B	Colorectal Cancer	139	166	59	140	162	50	0.08	0.78	Y
Antognelli et al.	2005	Caucasian	PCR-RFLP	H-B	Prostate Cancer	120	197	67	148	169	43	0.65	0.35	Y
Herrera et al.	2015	Mixed	TaqMan	H-B	Brain Tumor	46	17	4	42	14	2	0.37	0.56	Y
J. De Roos et al.	2006	Mixed	TaqMan	P-B	Hematologic Cancer	299	307	100	282	260	69	0.59	0.44	Y
Stevens et al.	2008	Mixed	TaqMan	P-B	Prostate Cancer	481	609	165	498	575	189	1.18	0.28	Y
Wang et al.	2012	Asian	PCR-RFLP	P-B	Lung Cancer	307	47	2	166	18	0	0.49	0.49	Y
Antognelli et al.	2013	Caucasian	PCR-RFLP	H-B	Prostate Cancer	180	291	100	497	540	131	0.75	0.39	Y
Hemati et al.	2019	Asian	PCR-RFLP	H-B	Gastric Cancer	41	40	9	34	49	7	0.027	0.87	Y

*Abbreviations*: PCR-RFlP, polymerase chain reaction-restriction fragment length polymorphism; HWE, Hardy–Weinberg equilibrium; Y, polymorphisms conforming to HWE in the control group; N, polymorphisms not conforming to HWE in the control group; H-B, hospital based; P-B, population based.

**Table 3:** Results of meta-analysis for PON1-Q192R polymorphism and cancer risk.

Variables	Case/control	R vs. Q			RR vs. QQ			RQ vs. QQ			RR+RQ vs. QQ			RR vs. RQ+QQ		
		OR(95% CI)	p^a^	I^2^(%)	OR(95% CI)	p^a^	I^2^(%)	OR(95% CI)	p^a^	I^2^(%)	OR(95% CI)	p^a^	I^2^(%)	OR(95% CI)	p^a^	I^2^(%)
Total	11412/13936	0.897(0.798-1.008)	0	86.8	0.855(0.683-1.073)	0	81.1	0.861(0.724-1.023)	0	86.7	0.857(0.730-1.008)	0	86.7	0.914(0.758-1.102)	0	78.1
Breast cancer	2005/2748	0.643(0.440-0.942) *∗*	0	91.4	0.542(0.331-0.886)*∗*	0	74	0.529(0.325-0.861)*∗*	0	89.1	0.534(0.330-0.865)*∗*	0	90.6	0.720(0.492-1.054)	0.010	62.3
Gastrointestinal cancer	1752/2356	1.008(0.700-1.450)	0	88.1	0.969(0.463-2.025)	0.000	80.2	1.079(0.761-1.529)	0.002	75.7	1.038(0.682-1.580)	0.000	85.0	0.968(0.547-1.711)	0.013	68.5
Prostate cancer	2261/2891	0.967(0.886-1.055)	0.748	0	0.563(0.313-1.015)	0.001	83	1.544(0.969-2.458)	0	90.9	1.249(1.030-1.514)*∗*	0.083	55	0.498(0.235-1.053)	0	90.3
Hematologic tumor	2303/2355	1.113(0.852-1.453)	0	85.5	1.358(0.787-2.341)	0	82	0.943(0.740-1.202)	0.007	62.3	1.040(0.774-1.397)	0	78.2	1.364(0.863-2.157)	0	77.8
Lung cancer	1172/1011	1.179(0.949-1.464)	0.04	63.8	1.244(0.665-2.326)	0.005	76.6	1.214(0.704-2.094)	0.004	77.5	1.262(0.741-2.149)	0.003	78.6	1.201(0.997-1.449)	0.442	0
Brain tumor	1173/1395	0.759(0.505-1.140)	0	89.2	0.576(0.266-1.248)	0	85.9	0.778(0.539-1.124)	0.006	69	0.696(0.431-1.123)	0	84.4	0.698(0.388-1.256)	0	79.4
Ovarian cancer	322/496	0.665(0.189-2.337)	0	92.7	0.940(0.314-2.813)	0.087	65.8	0.328(0.030-3.572)	0	94.6	0.443(0.060-3.283)	0	94.5	1.359(0.985-1.875)	0.658	0
Other cancers	424/684	0.809(0.481-1.362)	0	85.1	1.264(0.515-3.101)	0.022	65	0.670(0.250-1.798)	0	89.4	0.741(0.299-1.838)	0	89.1	1.352(0.797-2.294)	0.209	31.8
Ethnicities																
Caucasian	4424/6292	0.815(0.658-1.011)	0	90.1	0.784(0.516-1.193)	0	84.5	0.731(0.528-1.010)	0	91.1	0.744(0.557-0.993)*∗*	0	90.3	0.893(0.608-1.311)	0	83.5
Asian	3253/3629	0.840(0.840-1.244)	0	89.9	1.019(0.689-1.506)	0	78.5	1.020(0.779-1.337)	0	76.3	1.022(0.758-1.377)	0	83.0	1.052(0.850-1.303)	0.025	54.4
Mixed	3735/4015	0.981(0.877-1.098)	0.035	51.6	0.913(0.727-1.145)	0.062	46.2	1.012(0.873-1.174)	0.109	38.8	0.990(0.851-1.153)	0.057	47	0.929(0.770-1.119)	0.115	38.1
Control source																
Population based	6871/8354	0.849(0.717-1.004)	0	88.8	0.802(0.604-1.065)	0	79.1	0.793(0.638-0.984)*∗*	0	85	0.789(0.630-0.988)*∗*	0	87.9	0.920(0.749-1.130)	0	70.3
Hospital based	4309/5667	0.897(0.798-1.008)	0	85.1	0.948(0.655-1.374)	0	83.2	0.927(0.704-1.221)	0	87.3	0.923(0.726-1.175)	0	85.5	0.967(0.696-1.342)	0	83.3
Genotype method																
PCR-RFLP	5445/6900	0.884(0.735-1.064)	0	88.4	0.888(0.735-1.064)	0	88.4	0.815(0.607-1.094)	0	90.4	0.839(0.646-1.091)	0	89.4	0.938(0.692-1.271)	0	80.5
TaqMan	5967/7036	0.922(0.801-1.060)	0	83	0.834(0.622-1.117)	0	81.3	0.952(0.824-1.099)	0.001	61.1	0.906(0.762-1.078)	0	76.7	0.915(0.736-1.139)	0	73.5

Notes: *∗* statistically significant (P<0.05); P value^a^: P value of Q test for heterogeneity test; I^2^: 0%–25% means no heterogeneity, 25%–50% means modest heterogeneity, and 50% means high heterogeneity. Abbreviations: CI, confidence interval; OR, odds ratio; PCR-RFLP, polymerase chain reaction-restriction fragment length polymorphism.

**Table 4:** Results of meta-analysis for PON1-L55M polymorphism and cancer risk.

Variables	Case/control	M vs. L			MM vs. LL			ML vs. LL			ML+MM vs. LL			MM vs. ML+LL		
		OR(95% CI)	p^a^	I^2^(%)	OR(95% CI)	p^a^	I^2^(%)	OR(95% CI)	p^a^	I^2^(%)	OR(95% CI)	p^a^	I^2^(%)	OR(95% CI)	p^a^	I^2^(%)
Total	8565/9996	1.277(1.127-1.448)*∗*	0	81.6	1.507(1.205-1.885)*∗*	0	68.5	1.192(1.064-1.337)*∗*	0.001	50.6	1.288(1.120-1.480)*∗*	0	70.9	1.417(1.176-1.708)*∗*	0	64.2
Breast cancer	1882/1731	2.186(1.438-3.323) *∗*	0	92.5	3.215(1.756-5.886)*∗*	0	81.8	1.579(1.145-2.177) *∗*	0.01	69.9	2.110(1.397-3.188)*∗*	0	85.3	2.727(1.563-4.756)*∗*	0	81.9
Gastrointestinal cancer	1803/2495	1.111(0.898-1.375)	0.071	50.7	1.165(0.848-1.601)	0.988	0	1.097(0.794-1.515)	0.023	61.5	1.120(0.829-1.512)	0.032	59.0	1.185(0.881-1.594)	0.996	0
Prostate cancer	2259/2888	1.233(0.971-1.566)	0	84.3	1.496(0.876-2.556)	0	85.5	1.291(1.071-1.557)*∗*	0.144	44.6	1.341(1.024-1.756)*∗*	0.008	74.6	1.284(0.838-1.966)	0.002	80.5
Hematologic tumor	1259/1187	1.271(1.059-1.525)*∗*	0.121	52.7	1.514(1.178-1.946)*∗*	0.376	0	1.212(0.954-1.540)	0.172	43.1	1.299(1.017-1.661)*∗*	0.124	52	1.405(1.111-1.778)*∗*	0.622	0
Ovarian cancer	377/553	1.219(0.965-1.539)	0.450	0	1.208(0.648-2.253)	0.393	0	0.833(0.536-1.296)	0.579	0	0.952(0.623-1.454)	0.802	0	1.482(0.800-2.743)	0.316	13.2
Lung cancer	579/418	1.095(0.666-1.801)	0.104	62.2	0.781(0.344-1.771)	0.404	0	1.074(0.711-1.622)	0.215	34.8	1.089(0.670-1.767)	0.146	52.7	0.806(0.361-1.799)	0.432	0
Other cancers	406/724	0.932(0.753-1.155)	0.333	12.6	0.884(0.505-1.548)	0.215	31	0.907(0.682-1.206)	0.801	0	0.910(0.696-1.190)	0.625	0	0.930(0.584-1.481)	0.252	25.4
Ethnicities																
Caucasian	3616/4392	1.231(1.028-1.474)*∗*	0	83.8	1.737(1.519-1.986)*∗*	0	72	1.170(1.034-1.324)*∗*	0.199	22.4	1.334(1.215-1.465)*∗*	0	70.7	1.407(1.092-1.813)*∗*	0	67.8
Asian	2257/2667	1.604(1.089-2.363)*∗*	0	80.7	2.093(1.295-3.381)*∗*	0.441	0	1.550(0.995-2.417)	0.000	79.7	1.624(1.041-2.535)*∗*	0	80.8	1.967(1.238-3.125)*∗*	0.656	0
Mixed	2692/2937	1.177(1.004-1.379)*∗*	0.019	63.1	1.137(0.955-1.354)	0.088	47.8	1.112(0.953-1.297)	0.268	22.1	1.126(1.006-1.261)*∗*	0.165	36.3	1.262(0.957-1.665)	0.034	58.4
Control source																
Population based	5787/6158	1.325(1.085-1.618)*∗*	0	88.7	1.568(1.091-2.253)*∗*	0	81.9	1.275(1.051-1.548)*∗*	0.401	4.5	1.275(1.051-1.548)*∗*	0	75.7	1.503(1.110-2.034)*∗*	0	80.5
Hospital based	2778/3838	1.240(1.056-1.456)*∗*	0	68.9	1.531(1.199-1.955)*∗*	0.132	29.8	1.255(1.020-1.543)*∗*	0.000	62.3	1.288(1.120-1.480)*∗*	0	66.7	1.411(1.173-1.698)*∗*	0.324	11.6
Genotype method																
PCR-RFLP	4376/4698	1.243(1.053-1.466)*∗*	0	82.2	1.571(1.183-2.087)*∗*	0	70.1	1.164(1.033-1.311)*∗*	0.145	26.5	1.246(1.045-1.487)*∗*	0	69.3	1.483(1.167-1.884)*∗*	0	62.7
TaqMan	4189/5298	1.330(1.091-1.622)*∗*	0	79.5	1.309(0.988-1.735)	0.091	41.4	1.307(1.026-1.665)*∗*	0.001	71.5	1.370(1.073-1.748)*∗*	0	74.7	1.264(0.986-1.620)*∗*	0.05	48.5

Notes: *∗* statistically significant (P<0.05); P value^a^: P value of Q test for heterogeneity test; I^2^: 0%–25% means no heterogeneity, 25%–50% means modest heterogeneity, and 50% means high heterogeneity.

Abbreviations: CI, confidence interval; OR, odds ratio; PCR-RFLP, polymerase chain reaction-restriction fragment length polymorphism.

## References

[1] Bredberg A. (2011). Cancer: more of polygenic disease and less of multiple mutations? A quantitative viewpoint. *Cancer*.

[2] Ivanišević J., Kotur-Stevuljević J., Stefanović A. (2017). Association of paraoxonase 1 and oxidative stress with acute kidney injury in premature asphyxiated neonates. *Chemico-Biological Interactions*.

[3] Assis R., Arcaro C., Gutierres V. (2017). Combined effects of curcumin and lycopene or bixin in yoghurt on inhibition of LDL oxidation and increases in HDL and paraoxonase levels in streptozotocin-diabetic rats. *International Journal of Molecular Sciences*.

[4] Farinati F., Piciocchi M., Lavezzo E., Bortolami M., Cardin R. (2010). Oxidative stress and inducible nitric oxide synthase induction in carcinogenesis. *Digestive Diseases*.

[5] Hussein Y. M., Gharib A. F., Etewa R. L., ElSawy W. H. (2011). Association of L55M and Q192R polymorphisms in paraoxonase 1 (PON1) gene with breast cancer risk and their clinical significance. *Molecular and Cellular Biochemistry*.

[6] Eroglu M., Yilmaz N., Yalcinkaya S., Ay N., Aydin O., Sezer C. (2013). Enhanced HDL-cholesterol-associated anti-oxidant PON-1 activity in prostate cancer patients. *Kaohsiung Journal of Medical Sciences*.

[7] Brophy V. H., Jampsa R. L., Clendenning J. B., McKinstry L. A., Jarvik G. P., Furlong C. E. (2001). Effects of 5′ regulatory-region polymorphisms on paraoxonase-gene (PON1) expression. *American Journal of Human Genetics*.

[8] Aviram M., Hardak E., Vaya J. (2000). Human serum paraoxonases (PON1) Q and R selectively decrease lipid peroxides in human coronary and carotid atherosclerotic lesions: PON1 esterase and peroxidase-like activities. *Circulation*.

[9] Chen L., Lu W., Fang L. (2016). Association between L55M polymorphism in Paraoxonase 1 and cancer risk: a meta-analysis based on 21 studies. *OncoTargets and Therapy*.

[10] Zhang M., Xiong H., Fang L. (2015). Paraoxonase 1 (PON1) Q192R gene polymorphism and cancer risk: a meta-analysis based on 30 publications. *Asian Pacific Journal of Cancer Prevention*.

[11] Eom S., Yim D., Lee C. (2015). Interactions between paraoxonase 1 genetic polymorphisms and smoking and their effects on oxidative stress and lung cancer risk in a korean population. *Plos One*.

[12] Wang H., Li L., Ding L., Zhang Z., Pu C. (2012). Association of genetic polymorphisms in the paraoxonase 1 gene with the risk and prognosis of non-small cell lung cancer in Chinese Han population. *Journal of Investigative Medicine*.

[13] Vasconcelos G. M., Aguiar Alves Gonçalves B., Montalvão-de-Azevedo R. (2014). PON1 Q192R polymorphism (rs662) is associated with childhood embryonal tumors. *Molecular Biology Reports*.

[14] Lau J., Ioannidis J. P. A., Schmid C. H. (1997). Quantitative synthesis in systematic reviews. *Annals of Internal Medicine*.

[15] Higgins J. P. T., Thompson S. G. (2002). Quantifying heterogeneity in a meta-analysis. *Statistics in Medicine*.

[16] DerSimonian R., Laird N. (1986). Meta-analysis in clinical trials. *Controlled Clinical Trials*.

[17] Tobias A., Campbell M. J. (1999). Modelling influenza epidemics in the relation between black smoke and total mortality. A sensitivity analysis. *Journal of Epidemiology & Community Health*.

[18] Begg C. B., Mazumdar M. (1994). Operating characteristics of a rank correlation test for publication bias. *Biometrics*.

[19] Ahmed N. S., Shafik N. M., Elraheem O. A., Abou-Elnoeman S. A. (2015). Association of paraoxonase-1(Q192R and L55M) gene polymorphisms and activity with colorectal cancer and effect of surgical intervention. *Asian Pacific Journal of Cancer Prevention*.

[20] Akkız H., Kuran S., Akgöllü E. (2013). Effect of PON1 gene polymorphisms in Turkish patients with hepatocellular carcinoma. *Meta Gene*.

[21] Antognelli C., Del Buono C., Ludovini V. (2009). CYP17, GSTP1, PON1 and GLO1 gene polymorphisms as risk factors for breast cancer: an Italian case-control study. *BMC Cancer*.

[22] Antognelli C., Mearini L., Talesa V. N., Giannantoni A., Mearini E. (2005). Association of CYP17, GSTP1, and PON1 polymorphisms with the risk of prostate cancer. *The Prostate*.

[23] Antognelli C., Mezzasoma L., Mearini E., Talesa V. N. (2013). Glyoxalase 1-419C>a variant is associated with oxidative stress: implications in prostate cancer progression. *Plos One*.

[24] Cheng T. D., Makar K. W., Neuhouser M. L. (2015). Folate-mediated one-carbon metabolism genes and interactions with nutritional factors on colorectal cancer risk: Women's Health Initiative Observational Study. *Cancer*.

[25] De Roos A. J. (2006). Metabolic gene variants and risk of non-hodgkin's lymphoma. *Cancer Epidemiology Biomarkers & Prevention*.

[26] Ergen A., Kılıcoglu O., Ozger H., Agachan B., Isbir T. (2011). Paraoxonase 1 192 and 55 polymorphisms in osteosarcoma. *Molecular Biology Reports*.

[27] Gallicchio L., McSorley M. A., Newschaffer C. J. (2007). Body mass, polymorphisms in obesity-related genes, and the risk of developing breast cancer among women with benign breast disease. *Cancer Detection and Prevention*.

[28] Geng R., Chen Z., Zhao X. (2014). Oxidative stress-related genetic polymorphisms are associated with the prognosis of metastatic gastric cancer patients treated with epirubicin, oxaliplatin and 5-fluorouracil combination chemotherapy. *Plos One*.

[29] Gold L. S., De Roos A. J., Brown E. E. (2009). Associations of common variants in genes involved in metabolism and response to exogenous chemicals with risk of multiple myeloma. *Cancer Epidemiology*.

[30] Gonzalez-Herrera L., Gamas-Trujillo P. A., Medina-Escobedo G. (2015). The paraoxonase 1 Gene c.-108C>T SNP in the promoter is associated with risk for glioma in mexican patients, but not the p.L55M or p.Q192R polymorphisms in the coding region. *Genetic Testing and Molecular Biomarkers*.

[31] Hussein Y. M., Gharib A. F., Etewa R. L., ElSawy W. H. (2011). Association of L55M and Q192R polymorphisms in paraoxonase 1 (PON1) gene with breast cancer risk and their clinical significance. *Molecular and Cellular Biochemistry*.

[32] Kaya M. O., Sinan S., Güler Ö. Ö., Arslan O. (2015). Is there a relation between genetic susceptibility with cancer? A study about paraoxanase (PON1) enzyme activity in breast cancer cases. *Journal of Enzyme Inhibition and Medicinal Chemistry*.

[33] Kokouva M., Koureas M., Dardiotis E. (2013). Relationship between the paraoxonase 1 (PON1) M55L and Q192R polymorphisms and lymphohaematopoietic cancers in a Greek agricultural population. *Toxicology*.

[34] Lurie G., Wilkens L. R., Thompson P. J. (2008). Genetic polymorphisms in the paraoxonase 1 gene and risk of ovarian epithelial carcinoma. *Cancer Epidemiology Biomarkers & Prevention*.

[35] Martínez C., Molina J. A., Alonso-Navarro H., Jiménez-Jiménez F. J., Agúndez J. A., García-Martín E. (2010). Two common nonsynonymous paraoxonase 1 (PON1) gene polymorphisms and brain astrocytoma and meningioma. *BMC Neurology*.

[36] Metin Z. B., Aydin S., Unur M. (2013). Oral squamous cell carcinoma and serum paraoxonase 1. *The Journal of Laryngology & Otology*.

[37] Naidu R., Har Y. C., Taib N. A. M. (2010). Genetic polymorphisms of paraoxonase 1 (PON1) gene: Association between L55M or Q192R with breast cancer risk and clinico-pathological parameters. *Pathology & Oncology Research*.

[38] Nielsen S. S., Mueller B. A., De Roos A. J., A. Viernes H., Farin F. M., Checkoway H. (2005). Risk of brain tumors in children and susceptibility to organophosphorus insecticides: the potential role of paraoxonase (PON1). *Environmental Health Perspectives*.

[39] Stevens V. L., Rodriguez C., Pavluck A. L., Thun M. J., Calle E. E. (2006). Association of polymorphisms in the paraoxonase 1 gene with breast cancer incidence in the CPS-II nutrition cohort. *Cancer Epidemiology Biomarkers & Prevention*.

[40] Stevens V. L., Rodriguez C., Talbot J. T., Pavluck A. L., Thun M. J., Calle E. E. (2008). Paraoxonase 1 (PON1) polymorphisms and prostate cancer in the CPS-II nutrition cohort. *The Prostate*.

[41] Uluocak N., Atilgan D., Parlaktas B. S., Erdemir F., Ates O. (2017). A pilot study assessing the association between paraoxonase 1 gene polymorphism and prostate cancer. *Turkish Journal of Urology*.

[42] Uyar O., Kara M., Erol D., Ardicoglu A., Yuce H. (2011). Investigating paraoxonase-1 gene Q192R and L55M polymorphism in patients with renal cell cancer. *Genetics and Molecular Research*.

[43] Arpaci A., Görmüş U., Dalan B., Berkman S., Isbir T. (2009). Investigation of PON1192 and PON1 55 polymorphisms in ovarian cancer patients in Turkish population. *In Vivo*.

[44] Tomatir A. G., Pehlivan S., Sahin H. H., Balci S. O., Budeyri S., Pehlivan M. (2015). Q192R and L55M polymorphisms of paraoxonase 1 gene in chronic myelogenous leukemia and chronic lymphocytic leukemia. *Anticancer Reseach*.

[45] Kafadar A. M., Ergen A., Zeybek U., Agachan B., Kuday C., Isbir T. (2006). Paraoxonase 192 gene polymorphism and serum paraoxonase activity in high grade gliomas and meningiomas. *Cell Biochemistry & Function*.

[46] Agachan B., Yilmaz H., Ergen H. A., Karaali Z. E., Isbir T. (2005). Paraoxonase (PON1) 55 and 192 polymorphism and its effects to oxidant-antioxidant system in Turkish patients with type 2 diabetes mellitus. *Physiological Research*.

[47] De Aguiar Gonçalves B. A., Vasconcelos G. M., Thuler L. C. S., Andrade C., Faro A., Pombo-De-Oliveira M. S. (2012). NQO1 rs1800566 (C609T), PON1 rs662 (Q192R), and PON1 rs854560 (L55M) polymorphisms segregate the risk of childhood acute leukemias according to age range distribution. *Cancer Causes & Control*.

[48] Van Der Logt E. M. J., Janssen C. H. J. M., Van Hooijdonk Z. (2005). No association between genetic polymorphisms in NAD(P)H oxidase p22 phox and paraoxonase 1 and colorectal cancer risk. *Anticancer Reseach*.

[49] Kerridge I., Lincz L., Scorgie F., Hickey D., Granter N., Spencer A. (2002). Association between xenobiotic gene polymorphisms and non-Hodgkin's lymphoma risk. *British Journal of Haematology*.

[50] Wu J., Fang M., Zhou X., Zhu B., Yang Z. (2017). Paraoxonase 1 gene polymorphisms are associated with an increased risk of breast cancer in a population of Chinese women. *Oncotarget *.

[51] Lincz L. F., Kerridge I., Scorgie F. E., Bailey M., Enno A., Spencer A. (2004). Xenobiotic gene polymorphisms and susceptibility to multiple myeloma. *Haematologica*.

[52] Vecka M., Jachymova M., Vavrova L., Kodydkova J., Macasek J., Urbanek M. (2012). Paraoxonase-1 (PON1) status in pancreatic cancer: relation to clinical parameters. *Folia Biologica*.

[53] Öztürk O., Kağnici Ö. F., Öztürk T. (2009). 192R allele of paraoxanase 1 (PON1) gene as a new marker for susceptibility to bladder cancer. *Anticancer Reseach*.

[54] Aksoy-Sagirli P., Cakmakoglu B., Isbir T. (2011). Paraoxonase-1 192/55 polymorphisms and the risk of lung cancer in a Turkish population. *Anticancer Reseach*.

[55] Conesa-Zamora P., Ruiz-Cosano J., Torres-Moreno D. (2013). Polymorphisms in xenobiotic metabolizing genes (EPHX1, NQO1 and PON1) in lymphoma susceptibility: a case control study. *BMC Cancer*.

[56] Rajaraman P., Hutchinson A., Rothman N. (2008). Oxidative response gene polymorphisms and risk of adult brain tumors. *Neuro-Oncology*.

[57] Zhao P., Zhao L., Zou P. (2012). Genetic oxidative stress variants and glioma risk in a Chinese population: a hospital-based case–control study. *BMC Cancer*.

[58] Attar R., Atasoy H., Inal-Gültekin G. (2015). The effects of PON1 gene Q192R variant on the development of uterine leiomyoma in Turkish patients. *In Vivo*.

[59] Tang W., Liu J., Wang Y. (2017). Association between Paraoxonase 1 polymorphisms and risk of esophagogastric junction adenocarcinoma: A case-control study involving 2,740 subjects. *Oncotarget *.

[60] Hemati M., Mansourabadi A. H., Bafghi M. K., Moradi A. (2019). Association between paraoxonase-1 gene Q192R and L55M polymorphisms and risk of gastric cancer: a case-control study from Iran. *Nucleosides, Nucleotides and Nucleic Acids*.

[61] Ouerhani S., Ben Bahria I., Rouissi K., Cherni L. (2017). Distribution of xenobiotic metabolising enzyme genotypes in different Tunisian populations. *Annals of Human Biology*.

[62] De Roos A. J., Gold L. S., Wang S. (2006). Metabolic gene variants and risk of non-hodgkin's lymphoma. *Cancer Epidemiology Biomarkers & Prevention*.

[63] Uluocak N., Atilgan D., Parlaktas B. S., Erdemir F., Ates O. (2017). A pilot study assessing the association between paraoxonase 1 gene polymorphism and prostate cancer. *Türk Üroloji Dergisi/Turkish Journal of Urology*.

[64] Elkiran E. T., Mar N., Aygen B., Gursu F., Karaoglu A., Koca S. (2007). Serum paraoxonase and arylesterase activities in patients with lung cancer in a Turkish population. *BMC Cancer*.

[65] Akçay M. N., Polat M. F., Yilmaz I., Akçay G. (2003). Serum paraoxonase levels in pancreatic cancer. *Hepato-Gastroenterology*.

[66] Antognelli C., Del Buono C., Ludovini V. (2009). CYP17, GSTP1, PON1 and GLO1 gene polymorphisms as risk factors for breast cancer: an Italian case-control study. *BMC Cancer*.

[67] Gallicchio L., McSorley M. A., Newschaffer C. J. (2007). Body mass, polymorphisms in obesity-related genes, and the risk of developing breast cancer among women with benign breast disease. *Cancer Epidemiology*.

[68] Ferrè N., Camps J., Fernández-Ballart J. (2003). Regulation of serum paraoxonase activity by genetic, nutritional, and lifestyle factors in the general population. *Clinical Chemistry*.

[69] Delimaris I., Faviou E., Antonakos G., Stathopoulou E., Zachari A., Dionyssiou-Asteriou A. (2007). Oxidized LDL, serum oxidizability and serum lipid levels in patients with breast or ovarian cancer. *Clinical Biochemistry*.

[70] Lu W., Fang L., Xiong H. (2016). Association between L55M polymorphism in Paraoxonase 1 and cancer risk: a meta-analysis based on 21 studies. *OncoTargets and Therapy*.

